# Who and Where are the University of São Paulo Medical School Graduates?

**DOI:** 10.6061/clinics/2019/e1147

**Published:** 2019-09-10

**Authors:** Gustavo Rosa Gameiro, Leonardo Kenji Sakaue Koyama, Ana Luisa Ito Baptista da Cruz, Alex Jones Flores Cassenote, Aline Gil Alves Guilloux, Aluísio Augusto Cotrim Segurado, Mário César Scheffer

**Affiliations:** I Faculdade de Medicina FMUSP, Universidade de Sao Paulo, Sao Paulo, SP, BR; II Departamento de Gastroenterologia, Faculdade de Medicina FMUSP, Universidade de Sao Paulo, Sao Paulo, SP, BR; III Departamento de Medicina Preventiva, Faculdade de Medicina FMUSP, Universidade de Sao Paulo, Sao Paulo, SP, BR; IV Departamento de Molestias Infecciosas e Parasitarias, Faculdade de Medicina FMUSP, Universidade de Sao Paulo, Sao Paulo, SP, BR

**Keywords:** University of São Paulo, Medical Education, Medical School, Human Resources in Health

## Abstract

**OBJECTIVE:**

To evaluate the impact of a complex-care-based medical school in the context of the Brazilian health care system on students' career choices.

**METHODS:**

This was a retrospective cross-sectional study based on medical regulatory organization records. It included records for 7,419 physicians who graduated from FMUSP. Geographic data were analyzed using Kernel maps, and the statistical analysis was performed with SPSS^®^ version 24.0. A *p*-value less than 0.05 was considered significant.

**RESULTS:**

Of the 7,419 physicians, 68.6% (95% CI 67.5-69.7) were male, and 20.7% (95% CI 19.8%-21.7%) had no medical specialty, compared to 46.4% nationwide. Internal medicine and surgery-based specialties were more popular, accounting for 39.4% (95% CI 38.3%-40.5%) and 16.8% (95% CI 15.5%-17.6%) of our study group, compared to the Brazilian averages of 25.9% and 13.5%. Our graduates also had a higher probability of staying in São Paulo City, especially when born outside the city.

**CONCLUSION:**

We believe that FMUSP remains an interesting model for studying the impact of a highly specialized center on the education and career choices of medical students.

## INTRODUCTION

The Faculdade de Medicina da Universidade de São Paulo (FMUSP) has been central to the formation of leaders in the context of the Brazilian medical system. It currently has the largest hospital complex in Latin America, and it is responsible for the graduation of 175 physicians each year, who go on to assume high-level positions in their specialties. In addition, FMUSP is known for innovation in several areas; its graduates (MDs) have been responsible for many surgical innovations, including one of the first living donor liver transplants 1, the solution for the transposition of great vessels 2 and the first successful uterine transplant 3, among many others. All of this makes FMUSP one of the 100 best medical schools in the world 4.

In Brazil, the distribution of physicians across the national territory is heterogeneous. Despite the fact that there are approximately 450,000 professionals in the country (approximately 2.18 physicians per 1000 inhabitants), they are concentrated in large Brazilian cities, such as Vitória (12.27 physicians per 1000 inhabitants). In contrast, some states such as Maranhão have 0.87 physicians per 1000 inhabitants, demonstrating an uneven distribution of medical professionals. This distribution has led the government to invest in programs that facilitate the creation of medical schools 5. This has contributed to a change in the profile of physicians, leading the population of physicians to become much younger, with a higher proportion of females, despite the male majority in the Brazilian population (54.4% male *vs*. 45.6% female) 6. The aim of this paper was to analyze the profile and distribution of graduates from FMUSP.

## MATERIALS AND METHODS

This study was a retrospective study based on databases, including the administrative and legal register from the Regional Medicine Councils (CRMs), the register of the Federal Medicine Council (CFM), the database from the National Committee of Medical Residency, and the database of the Brazilian Medical Association. These databases combine data from all medical specialty societies, and the filing of their information is mandatory for medical practitioners.

We included 7,419 records from physicians who had graduated from FMUSP between 1940 and 2013 and who were 21 to 75 years old. Duplicated or incomplete registry entries were excluded from the present study.

In our analysis, we divided all graduates in 53 medical specialties into the following three main areas:

1. **Internal medicine-based specialties:** Acupuncture, Allergy and Immunology, Angiology, Cardiology, Internal Medicine, Dermatology, Gastroenterology, Endocrinology and Metabolism, Endoscopy, Medical Genetics, Geriatric Medicine, Hematology and Hemotherapy, Homeopathy, Infectious Disease, Family and Community Medicine, Occupational Medicine, Traffic Medicine, Sports Medicine, Physical Medicine and Rehabilitation, Intensive Medicine, Legal Medicine, Nuclear Medicine, Social and Preventive Medicine, Neurology, Nutrition, Pathology, Clinical Pathology and Laboratory Medicine, Pediatrics, Pneumology, Psychiatry, Radiology and Diagnostic Imaging, Radiotherapy, Rheumatology;
2. **Surgery-based specialties**: Anesthesiology, Cardiovascular Surgery, Hand Surgery, Head and Neck Surgery, Digestive Tract Surgery, General Surgery, Pediatric Surgery, Plastic Surgery, Thoracic Surgery, Vascular Surgery, Neurosurgery;
3. **Both**: Cancerology, Coloproctology, Obstetrics and Gynecology, Mastology, Nephrology, Ophthalmology, Orthopedics and Traumatology, Otorhinolaryngology and Urology.

A review of historical documents, loaned by the Carlos da Silva Lacaz Historical Museum at FMUSP, allowed the collection of the data needed for the analysis of the years included in this study.

As an exploratory analysis, we plotted two Kernel density maps using a qualitative geographic information system open source software (QGis) for MDs who were FMUSP graduates and who were born either inside or outside of São Paulo City. This technique allows us to better visualize the overlap and proximity of the current work locations of the MDs via a color scale and visual analysis. Both maps have the same bandwidth, and their categories have the same breakpoint values to allow a better visual comparison.

This study was part of the “Brazilian Medical Demography: Profile, Distribution, Work and Specialization of Physicians” Project, approved by the FMUSP Ethics in Research Committee (Resolution 797.424 on 09/03/2014).

The authors then analyzed the data obtained, identifying correlations between these data and data from other medical demography studies. All data were analyzed using appropriate statistical methods with IBM 24.0 SPSS^®^ software. A *p*-value less than 0.05 was considered statistically significant.

## RESULTS

**Figure 1:**
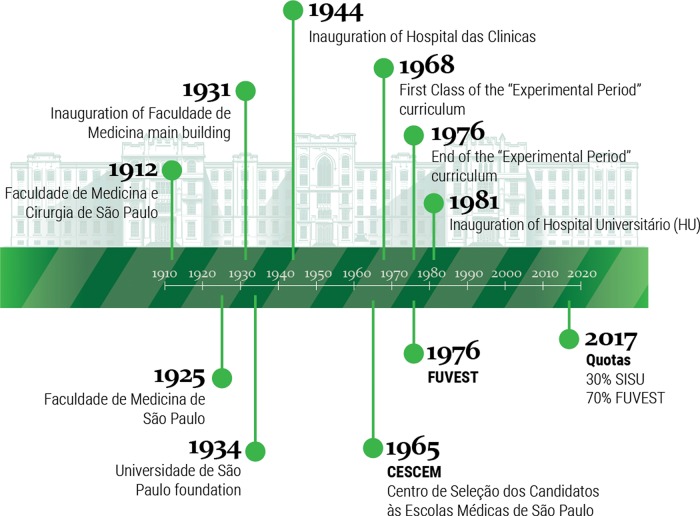
Important landmarks in USP Medical School history. Please note that CESCEM is an institution created to select candidates to enter medical schools in the state of São Paulo (Brazil). “Quotas” is used to refer to a Brazilian governmental program of affirmative action.

The timeline (Figure 1) shows events in the history of FMUSP that may have directly impacted the formation of the profile of medical students there. It was founded in 1912 as Faculdade de Medicina e Cirurgia de São Paulo. In 1916, it received financial support from São Paulo's state government and from the Rockefeller Foundation, with the purpose of initiating a new medical school model based on teaching hospitals. In 1944, the Central Institute (IC) of the Hospital das Clínicas (HC) was founded. Today, it is a referral center for complex cases from across Brazil.

There are currently 7,419 physicians who are FMUSP graduates and who are younger than 70 years, accounting for approximately 1.7% of all currently working physicians in Brazil (432,870 in total) in 2015. Since the foundation of FMUSP in 1912 until 1976, the number of graduating MDs has gradually increased each year. Today, 175 students receive a medical degree from FMUSP annually, a number that is approximately 6 times larger than that of the first class in 1918. The demographic data of the FMUSP graduates are presented in Table 1.

We can observe that 68.6% of the physicians who have graduated from FMUSP are men. This is significantly different from the general profile of Brazilian physicians, 57.5% of whom are women. However, the proportion of females has grown impressively grown since the founding of FMUSP; it barely rose above 15% in the first 6 decades, reaching 24.5% in the 7^th^ decade, 37.5% on the 8^th^ decade and stabilizing approximately 37% on the following decades.

Concerning the medical specialties chosen by FMUSP graduates, there is a higher percentage of specialists in all areas among FMUSP graduates than among physicians in Brazil as a whole, according to the 2015 Medical Demography database. In total, 1,537 graduates do not have a specialty registered or it was not possible to access it (20.7% of the study group). The specialties with the largest numbers of graduates are General Surgery with 826 physicians (10.4%), Pediatrics with 594 (7.5%), Internal Medicine with 565 (7.1%), Gynecology and Obstetrics with 461 (5.8%), Orthopedics and Traumatology with 451 (5.7%), Psychiatry with 378 (4.8%) and Radiology with 359 (4.5%). According to the Brazilian Medical Demography database, among the specialists, 25.9% are in internal medicine, 13.5% are in surgical areas and 14.2% are in both (internal medicine and surgery-based). Table 2 shows all the specialties in detail.

FMUSP graduates and all Brazilian physicians have similar profiles in terms of the age group distribution of graduates aged 35 years or more. However, it has proportionally fewer young physicians (younger than 35 years old) when compared to national physician population.

**Figure 2:**
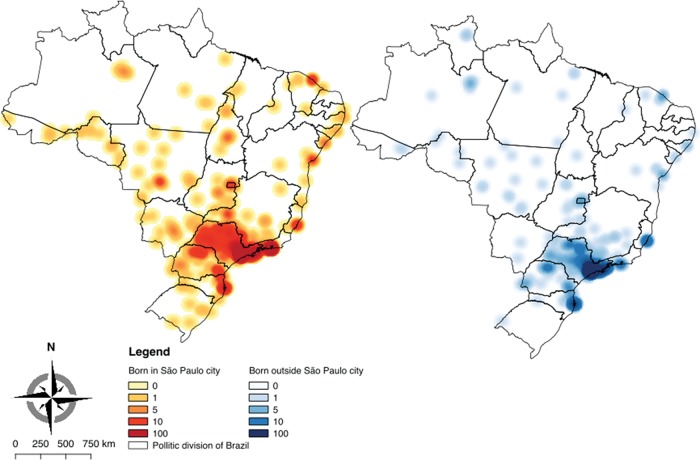
**Kernel** maps showing the geographic distribution of physicians who graduated from FMUSP.

Table 3 shows that most of the graduates of FMUSP were born in São Paulo State (83.2%), followed by those born in neighboring states, like Minas Gerais, Rio de Janeiro and Paraná, as seen in Figure 2. In total, 94.5% of the students were born in one of the Southeast States (São Paulo, Rio de Janeiro, Minas Gerais or Espírito Santo), and most of the students were born in São Paulo City, where FMUSP is located. Students who were not born in Brazil account for a very small portion of graduates (0.4%).

The maps in Figure 2 show the geographic distribution of FMUSP graduates across Brazil; the graduates are divided into two groups, namely, those who were born in São Paulo City and those who were born outside of São Paulo City. Another characteristic of FMUSP graduates is the tendency to stay in São Paulo City. While 55.6% were born outside the city, 75.2% of FMUSP graduates practice medicine in São Paulo, resulting in an even larger concentration of medical professionals in the area.

## DISCUSSION

Over the 105 years of its history, FMUSP has occupied an important position in the formation of the Brazilian medical system. Being a pioneer makes it a global model in several areas 7 and one of the main healthcare education centers and the most important research center in the Health Sciences in Brazil 8.

It is curious to note that in the last three decades, the percentage of women at FMUSP has remained approximately 37.5%. This does not reflect Brazilian reality; in recent years, there has been an increasing proportion of female physicians graduating compared to their male counterparts, a proportion that is similar to that found in several medical schools around the world 9. In Brazil, since 2011, female physicians account for the majority of registered physicians 10.

Another noteworthy finding is that among FMUSP graduates, 33.6% do not have any registered medical specialty. This could reflect mainly an inefficiency in updating the database because this information is not mandatory. Furthermore, the average of unregistered specialties is still lower than the national average (46.4%) 6. Therefore, FMUSP still produces more specialists than the average Brazilian medical school. We believe this is related to the fact that the associated teaching hospital is a tertiary center with a concentration of highly complex cases.

The HC is the teaching hospital throughout the 6 years of education for FMUSP students. The hospital includes specialized institutes that focus on highly complex cases. The effect of a highly specialized center on medical training is still unknown. Sierles and Taylos have demonstrated a correlation between medical education and professional choices 11. Career choices directly affect the available medical workforce 12; therefore, it has the potential to affect health policy planning.

There are substantial differences between the most common specialties in several countries, with various conditions linked to historical events in each country studied 13,14. The diversity of career choices can also be linked to all the medical specialties offered by the Hospital das Clínicas (HC) / FMUSP complex, the Medical Residency programs of which are attended by a large part of the institution's alumni.

Moreover, we observed a higher percentage of FMUSP graduates who were specialists compared with the general population of physicians in Brazil. Another interesting finding was the slightly higher prevalence of surgical specialties as the chosen career among FMUSP graduates compared to the national average [16.8% (95% CI 15.5%-17.6) *versus* 13.5%]. We believe that is related to the international recognition of and pioneering work performed by our institution's surgical groups 7.

It is notable, however, that a low percentage of our MDs go on to work in areas such as Family Medicine, Pediatrics and Obstetrics and Gynecology, which are essential to primary care and have direct impacts on public policy. We believe that these choices are related to the tertiary hospital in which the students practice and that this model is essential for wellbeing of the nation. Although FMUSP does not generate as many primary care physicians, it educates highly specialized individuals who are also important for the development of health care in Brazil. However, few studies have compared the level of the teaching hospital with the choices of specialties made by graduates.

In addition, the Kernel map shows one more interesting feature of FMUSP graduates; the students who come from other cities outside of São Paulo City tend to stay in São Paulo more often than do those who were born in the city. This phenomenon is shown in Table 4 and could be related to factors such as the greater size of the population (meaning that there would be a larger pool of patients for their chosen specialty), more modern technological resources, a known network, a larger number of hospitals and activity fields, as well as the possibility of maintaining a bond with the university 15,16. This disparity in the distribution of graduates agrees with the high percentage of physicians in São Paulo City, which has 23.7% of all physicians in the Southeast Region and 12.7% of all physicians in Brazil.

In 2013, the “Mais Médicos” law was promulgated in Brazil (law 12.871 on October 22, 2013), which stimulates the creation of new medical schools to provide more physicians for the Brazilian population. The law was highly criticized, with many suggesting that the focus should be on a better distribution of the existing physicians rather than on the training of additional physicians. The attachment of our students to the university's city, as previously observed by other authors, supports the critics of this law, as most of the new medical schools were created in the São Paulo region, whereas regions with fewer physicians per inhabitant such as Maranhão e Pará gained no new medical schools 17,18. It is our belief, therefore, that the law only contributes to a less equal distribution of Brazilian physicians.

## CONCLUSIONS

We believe that the characteristics of FMUSP may guide decisions related to public health and educational policies. The analysis of the century-long experience of FMUSP and its graduates allow us to make predictions about the outcomes of actions directed at improving public health.

As FMUSP has recently adopted affirmative action and racial quotas, we believe that our student profile will undergo even greater changes in the future. However, we believe that FMUSP remains an interesting model for the impact of a highly specialized center on the education and choices of medical students.

### Limitations

Since this study is based on secondary data from different databases that are not always updated, it is expected that some data may be missing. Nevertheless, it was possible to determine a profile of the professionals who graduated from one of the largest medical education centers in Latin America, including their specialization. The database used in this study is, to the best of our knowledge, the best register of medical professionals in our country. In the future, these data could help establish health policy to foster medical education and to foment discussions on this topic.

## AUTHOR CONTRIBUTIONS

Gameiro GR, Koyama LKS, Cruz ALIB and Cassenote AJF contributed to the manuscript conception and design, literature review, statistical analysis, manuscript drafting, critical revision and final approval. Guilloux AGA contributed to the statistical analysis and manuscript final approval. Segurado AAC and Scheffer MC contributed to the manuscript conception and design, manuscript critical revision and final approval.

## Figures and Tables

**Table 1:** The statistical description of physicians who graduated from FMUSP and their specialties. The percentages presented correspond to the total number of physicians and the total number of specialists, respectively.

	N*	Frequency (%)	95%CI	%MD**
**Gender**				
Male	5,087	68.6	(67.5-69.7)	57.5
Female	2,332	31.4	(30.3-32.5)	42.5
**Specialties**				
No specialty	1,537	20.7	(19.8-21.7)	46.4
Internal Medicine-Based	2,921	39.4	(38.3-40.5)	25.9
Surgery-Based	1,243	16.8	(15.5-17.6)	13.5
Both	1,718	23.2	(22.2-24.1)	14.2
**Age (Grouped by 5 Years)**				
≤29	657	8.8	(8.2-9.5)	14.3
30–34	848	11.4	(10.7-12.2)	15.0
35–39	835	11.2	(10.5-12.0)	12.2
40–44	731	9.8	(9.1-10.5)	9.6
45–49	796	10.7	(10.0-11.4)	8.9
50–54	743	10.0	(9.4-10.7)	9.0
55–59	751	10.1	(9.4-10.8)	9.1
60–64	692	9.3	(8.7-10.0)	9.2
65–69	392	5.3	(4.7-5.8)	5.9
≥70	974	13.1	(12.3-13.9)	6.9
**Region of Birth**				
Northern	26	0.4%	(0.3-1.3)	
Northeastern	93	1.4%	(0.9-2.3)	
Southeastern	6,476	94.5%	(92.4-94.6)	
Southern	164	2.4%	(2.0-3.1)	
Central-western	94	1.4%	(1.3-2.3)	
**Region of Medical Registry**				
Northern	76	1%	(0.2-0.6)	4.4
Northeastern	95	1.3%	(0.5-2.4)	17.4
Southeastern	6,934	93.5%	(93.0-96.1)	55.3
Southern	185	2.5%	(1.8-3.0)	15.0
Central-western	129	1.7%	(0.8-2.3)	7.9
**Total**	7,419	100%		

* May be missing data in some dataset variables.

** Distribution in the population-Demografia Médica no Brasil (8).

**Table 2:** Medical specialties of physicians who graduated from the USP Medical School.

Specialties	N*	%	%MD**	Diff
General Surgery	826	10.4	8.8	1.6
Pediatrics	595	7.5	10.5	-3.0
Internal Medicine	565	7.1	10.6	-3.5
Obstetrics & Gynecology	461	5.8	8.6	-2.8
Orthopedics & Traumatology	451	5.7	4.0	1.7
Psychiatry	378	4.8	2.7	2.1
Radiology and Diagnostic Imaging	359	4.5	2.9	1.6
Anesthesiology	334	4.2	6.3	-2.1
Dermatology	260	3.3	2.0	1.3
Ophthalmology	254	3.2	3.5	-0.3
Cardiology	244	3.1	4.0	-0.9
Otorhinolaryngology	215	2.7	1.7	1.0
Plastic Surgery	202	2.5	1.7	0.8
Urology	177	2.2	1.4	0.8
Intensive Medicine	165	2.1	1.5	0.6
Digestive Tract Surgery	153	1.9	0.7	1.2
Endocrinology and Metabolism	134	1.7	1.3	0.4
Neurology	134	1.7	1.3	0.4
Occupational Medicine	132	1.7	4.0	-2.3
Acupuncture	116	1.5	0.9	0.6
Vascular Surgery	105	1.3	1.0	0.3
Pathology	105	1.3	0.9	0.4
Pneumology	91	1.1	0.9	0.2
Neurosurgery	90	1.1	0.8	0.3
Nephrology	81	1.0	1.1	-0.1
Infectious Diseases	78	1.0	0.9	0.1
Geriatric Medicine	71	0.9	0.4	0.5
Head and Neck Surgery	71	0.9	0.2	0.7
Social and Preventive Medicine	69	0.9	0.5	0.4
Medicine of Traffic	69	0.9	1.1	-0.2
Endoscopy	65	0.8	0.8	0.0
Hematology and Hemotherapy	64	0.8	0.7	0.1
Rheumatology	59	0.7	0.6	0.1
Physical Medicine & Rehabilitation	58	0.7	0.2	0.5
Family and Community Medicine	54	0.7	1.2	-0.5
Cardiovascular Surgery	50	0.6	0.6	0.0
Cancerology	48	0.6	1.0	-0.4
Clinical Pathology / Laboratory Medicine	47	0.6	0.5	0.1
Gastroenterology	47	0.6	1.3	-0.7
Mastology	45	0.6	0.5	0.1
Hand Surgery	42	0.5	0.1	0.4
Thoracic Surgery	39	0.5	0.2	0.3
Homeopathy	38	0.5	0.7	-0.2
Nuclear Medicine	38	0.5	0.2	0.3
Coloproctology	37	0.5	0.5	0.0
Sports Medicine	37	0.5	0.2	0.3
Allergy & Immunology	35	0.4	0.4	0.0
Nutrition	35	0.4	0.4	0.0
Angiology	34	0.4	0.5	-0.1
Pediatric Surgery	32	0.4	0.3	0.1
Radiotherapy	23	0.3	0.1	0.2
Legal Medicine	15	0.2	0.2	0.0
Medical Genetics	5	0.1	0.0	0.1
	7,932	100.0		

* The number of specialties is higher than the number of physician because 1 physician can have more than one specialty.

** Distribution in the population - Demografia Médica no Brasil 8.

**Table 3:** States where graduates of FMUSP were born and are registered.

State	Birth (n)	Percentage	Registered (n)	Percentage
Acre	3	0.0	6	0.1
Alagoas	6	0.1	9	0.1
Amapá	1	0.0	-	-
Amazonas	9	0.1	20	0.3
Bahia	28	0.4	32	0.4
Ceará	16	0.2	31	0.4
Distrito Federal	13	0.2	49	0.7
Espírito Santo	14	0.2	13	0.2
Goiás	34	0.5	22	0.3
Maranhão	7	0.1	2	0.0
Mato Grosso	12	0.2	37	0.5
Mato Grosso do Sul	35	0.5	21	0.3
Minas Gerais	159	2.1	73	1.0
Pará	10	0.1	16	0.2
Paraíba	7	0.1	4	0.1
Paraná	111	1.5	111	1.5
Pernambuco	16	0.2	5	0.1
Piauí	4	0.1	3	0.0
Rio de Janeiro	127	1.7	59	0.8
Rio Grande do Norte	2	0.0	-	-
Rio Grande do Sul	37	0.5	10	0.1
Rondônia	3	0.0	18	0.2
Roraima	-	-	2	0.0
Santa Catarina	16	0.2	64	0.9
São Paulo	6176	83.2	6797	91.5
Sergipe	7	0.1	9	0.1
Tocantins	-	-	14	0.2
Total	6853	92.3	7427	100

**Table 4:** Analysis of professionals who were born in São Paulo (city) *versus* those who are born outside São Paulo (city).

Chi-square <0.001		Born inside São Paulo (city)	Born outside São Paulo (city)	Total
**Live in São Paulo (city)**	Count	1999	3396	5395
	% Table	27.9%	47.3%	75.2%
	% Column	62.7%	85.1%	
	Remainder	-5.52%	5.52%	
**Live outside São Paulo (city)**	Count	1187	595	1782
	% Table	16.5%	8.3%	24.8%
	% Column	37.3%	14.9%	
	Remainder	5.52%	-5.52%	
**Total**	Count	3186	3991	7177
	% Table	44.4%	55.6%	100%
